# Clinical features and outcomes of duodenal‐type follicular lymphoma: A single‐center retrospective study

**DOI:** 10.1002/jha2.384

**Published:** 2022-01-31

**Authors:** Kimimori Kamijo, Yoshimitsu Shimomura, Satoshi Yoshioka, Daisuke Yamashita, Shigeo Hara, Takayuki Ishikawa

**Affiliations:** ^1^ Department of Hematology Kobe City Hospital Organization Kobe City Medical Center General Hospital Chuo‐ku Japan; ^2^ Department of Environmental Medicine and Population Science Graduate School of Medicine Osaka University Suita Japan; ^3^ Department of Pathology Kobe City Hospital Organization Kobe City Medical Center General Hospital Chuo‐ku Japan

**Keywords:** duodenal‐type follicular lymphoma, esophagogastroduodenoscopy, follicular lymphoma, spontaneous complete regression, watch and wait strategy

## Abstract

Duodenal‐type follicular lymphoma (FL) is a rare and newly recognized disease. Few data are available on the outcomes and treatment strategies for patients with duodenal‐type FL. We aimed to investigate the clinical features and outcomes of duodenal‐type FL. We defined duodenal‐type FL as involvement of the duodenum, without nodal or extranodal lesions other than intestinal lesions, pathologically diagnosed as FL. We reviewed 26 patients with duodenal‐type FL between January 2011 and December 2020 at Kobe City Hospital Organization, Kobe City Medical Center General Hospital. In particular, patients were selected for the watch and wait (WW) strategy and followed up with regular esophagogastroduodenoscopy about once a year at our institution. The patient characteristics were as follows: median age 63.5 years (range: 42–78), sex (male, 15; female, 11), stage (I, 26), and grade (I, 26). Regarding treatment strategies, 23 patients were selected for the WW strategy, and three patients received initial rituximab therapy. The median follow‐up period was 65.5 months (range: 0.2–109). Five‐year progression‐free survival and 5‐year overall survival rates were 86.3% and 100%, respectively. Among the 23 patients selected for the WW strategy, six had spontaneous complete regression, and 14 had stable disease, and three had progressive disease, including one with histologic transformation. The WW strategy for patients with duodenal‐type FL could be an appropriate and safe treatment option. However, in several cases, disease progression was documented, and regular follow‐up is important.

## 1 INTRODUCTION

Follicular lymphoma (FL) is the most common form of indolent non‐Hodgkin lymphoma, defined as a neoplasm of germinal‐center B cells, usually with a follicular growth pattern [1]. FL commonly presents with an advanced stage with widespread nodal disease and eventual secondary involvement of extranodal sites [2]. Conversely, limited‐stage FL is an uncommon entity [3]. Although patients who present with advanced disease are often considered incurable, approximately half of the patients with the limited disease have long‐term remission, usually following treatment with radiation therapy [2, 3].

Duodenal‐type FL (DFL), which was recognized in the 2017 World Health Organization classification, is a rare and specific variant of FL that is predominantly involved in the second portion of the duodenum [4]. In general, DFL has a very indolent clinical course and an excellent prognosis but rarely progresses, including histologic transformation (HT) [4, 5, 6, 7, 8]. Although the watch and wait (WW) strategy is frequently applied, there is no consensus regarding the treatment [9]. In addition, fewer data are available on the outcomes of patients with DFL since DFL is a rare and newly recognized disease.

Therefore, we followed up patients with DFL with regular esophagogastroduodenoscopy (EGD) about once a year and aimed to investigate the clinical features and outcomes of DFL.

## 2 MATERIAL AND METHODS

### 2.1 Study patients

In this study, we defined DFL as involvement of the duodenum, without nodal or extranodal lesions other than intestinal lesions, pathologically diagnosed as FL.

We retrospectively identified 350 patients with newly diagnosed FL at our institution between January 2011 and December 2020. We found that 36 patients with FL had duodenal involvement. Among them, a total of 10 patients were excluded for the following reasons: (i) nodal or extranodal lesions other than intestinal lesions (*n* = 9); and (ii) unavailable EGD data on initial examination (*n* = 1). The remaining 26 patients were included in the study.

The study protocol complied with the Helsinki Declaration standards and was approved by the Ethical Committee of Kobe City Hospital Organization, Kobe City Medical Center General Hospital (approval number: 21032). The requirement for written informed consent was waived as this study used retrospective data obtained from hospital records, and there were no interventions in the study patients.

### 2.2 Routine analysis

The diagnostic work‐up and staging procedures on presentation included inquiry of patient's medical history and complete physical examination, chest radiography, whole‐body computed tomography (CT) scanning, and/or (18)F‐fluorodeoxyglucose positron emission tomography/CT ((18)F‐FDG PET/CT), and bone marrow examination. The clinical staging of each patient was determined according to the Lugano classification of gastrointestinal lymphoma [10]. In most patients, video capsule endoscopy or double‐balloon endoscopy was performed as an additional test. Regarding follow‐up strategy, physical examination and blood tests were performed every 3–6 months, and CT and EGD were performed every 12 months in all patients. The diagnostic test and follow‐up strategy tended to follow the above‐mentioned principle however were eventually determined by each attending physician.

### 2.3 Definition

At the time of diagnosis of DFL, complaining of gastrointestinal symptoms regardless of their degree was defined as symptomatic. In patients who received initial treatment, assessment of response was determined according to the Lugano classification [11]. In patients who were selected for the WW strategy, the following definitions were used for evaluation: spontaneous complete regression (sCR) was defined as complete disappearance of the lesion or slight persistence of the lesion, but negative results were confirmed by two or more biopsies by EGD; progressive disease was defined as an enlargement of the tumor or the appearance of new tumor lesions; stable disease (SD) was defined as neither sCR nor PD; overall survival (OS) was measured from the date of diagnosis to the date of death or the last follow‐up used for censoring; progression‐free survival (PFS) was measured from the date of diagnosis to the date of progression. One patient who underwent total gastrectomy for gastric cancer and was unable to undergo EGD was censored at the time of gastrectomy.

### 2.4 Statistical analyses

All variables shown in the table and the figures were retrospectively obtained from the patient records. Continuous variables were summarized using medians with interquartile ranges (quartiles 1–3), and categorical variables were summarized as counts and percentages. Event rates of OS and PFS were estimated using the Kaplan–Meier method with a 95% confidence interval. All statistical analyses were performed using EZR (Saitama Medical Center, Jichi Medical University, Saitama, Japan) [12].

## 3 RESULTS

The patient characteristics at diagnosis are summarized in Table 1. The median age was 63.5 years (range: 42–78), and 15 (58%) patients were male. While 20 (77%) patients were asymptomatic, six (23%) patients had abdominal symptoms that led to EGD, abdominal pain (*n* = 3), and abdominal discomfort (*n* = 3). Findings of EGD revealed macroscopic characteristics and locations as follows: 21 (81%) multiple whitish small polyps (only second portion [*n* = 16], only third portion [*n* = 1], second plus third portion [*n* = 4]), three (11%) flat‐elevated lesion (only second portion [*n* = 3]), one (4%) semicircumferential redness (only second portion [*n* = 1]), and one (4%) depressed superficial lesion (only second portion [*n* = 1]) in the duodenum (Table 2). In addition, 21 patients (81%) underwent video capsule endoscopy or double‐balloon endoscopy. Besides the duodenal lesions, other small bowel lesions were found in 19 (73%) patients; nine had lesions in the jejunum, two had lesions in the ileum, and eight had lesions in both. (18)F‐FDG PET/CT was performed in 22 patients (85%). Among them, the duodenum lesion was positive on (18)F‐FDG PET/CT in seven (27%) patients. All patients were classified as stage I according to the Lugano classification and low risk as per the international prognostic index and follicular lymphoma international prognostic index. The histological grade of FL was grade 1 in all 26 patients (100%).

**TABLE 1:** Duodenal‐type follicular lymphoma patient characteristics

Number of patients	*N* = 26	%
Age (years)		
Median (range)	63.5 (42–78)	
Sex		
Female	11	42
Male	15	58
Clinical symptoms		
Present	6	23
Absent	20	77
Sites of lesions in the duodenum		
Bulbs	0	0
Second portion only	21	81
Third portion only	1	4
Second and third portion	4	15
Lesions in other regions of small intestine		
Present	19	73
Absent	7	27
Stage (Lugano's classification)		
Ⅰ	26	100
Ⅱ_1_	0	0
Ⅱ_2_	0	0
IV	0	0
IPI		
Low risk	26	100
FLIPI		
Low risk	26	100
Histological grade		
Grade 1	26	100
Immunostaining		
CD20 +	26	100
CD10 +	24	92
BCL2 +	26	100

Abbreviations: FLIPI, follicular lymphoma international prognostic index; IPI, international prognostic index.

**TABLE 2:** Esophagogastroduodenoscopy findings and anatomical locations of duodenal‐type follicular lymphoma

	Multiple whitish small polyps	Flat‐elevated lesion	Semicircumferential redness	Depressed superficial lesion
2nd	16	3	1	1
3rd	1	0	0	0
2nd and 3rd	4	0	0	0

*Note*: 2nd: second portion of the duodenum; 3rd: third portion of the duodenum.

**FIGURE 1:**
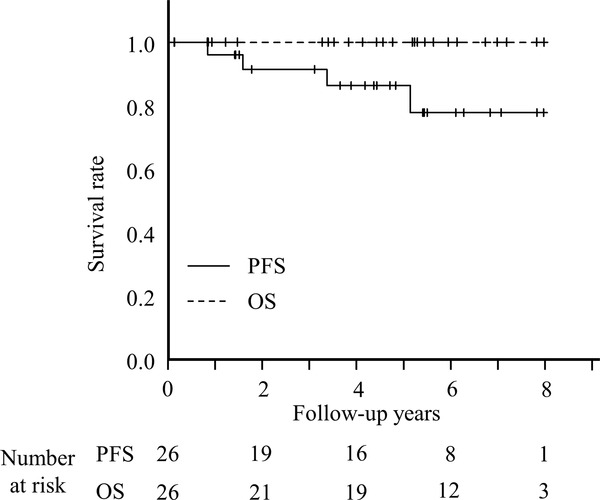
Progression‐free survival and overall survival of all patients. OS, overall survival; PFS, progression‐free survival

**FIGURE 2:**
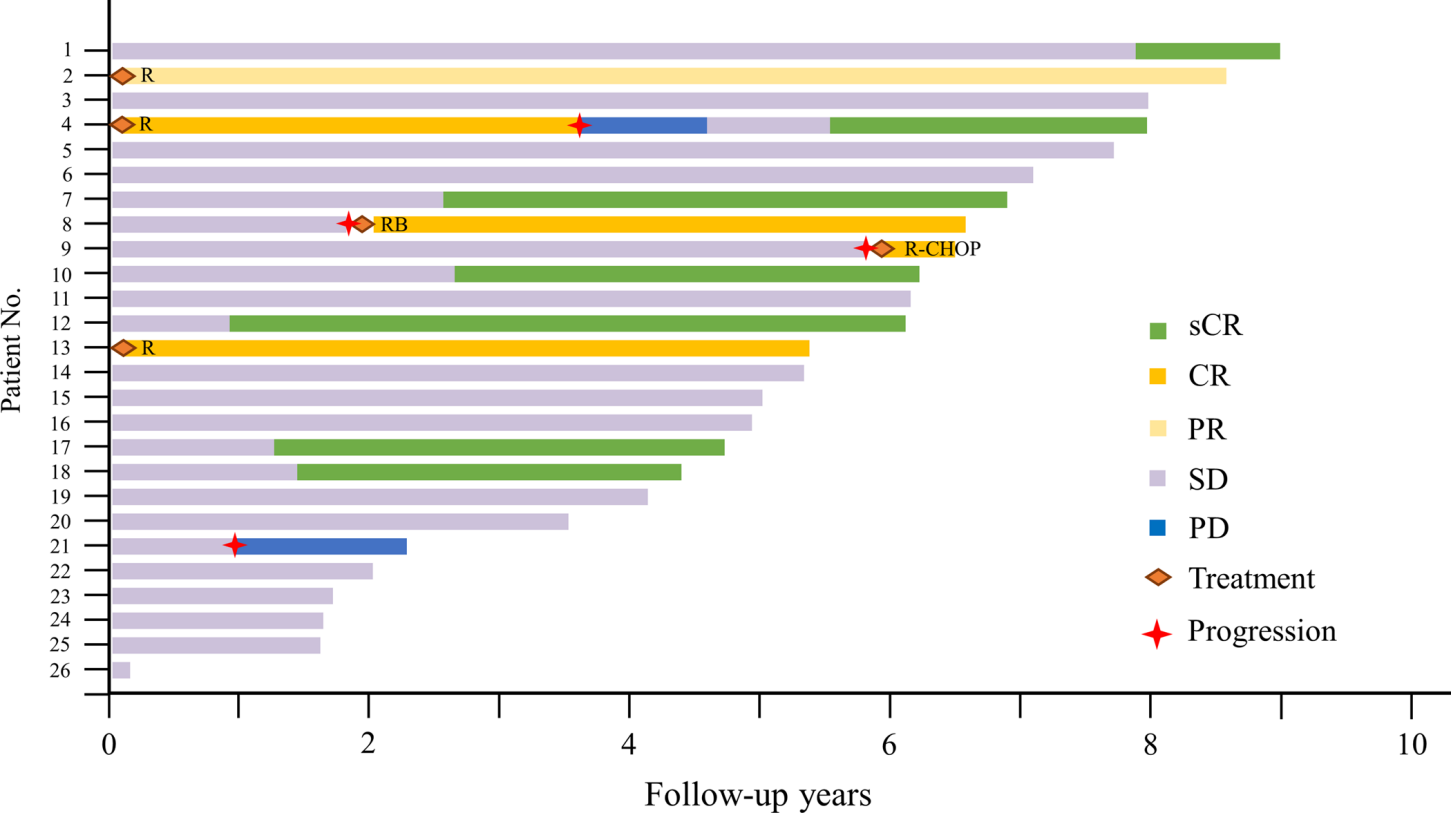
Summary of the clinical course of 26 duodenal‐type follicular lymphoma patients. CHOP, cyclophosphamide, doxorubicin, vincristine, and prednisolone; CR, complete response; PD, progressive disease; PR, partial response; R, rituximab; RB, rituximab, bendamustine; sCR, spontaneous complete regression; SD, stable disease

Over the median follow‐up period of 65.5 months (range: 0.2–109), 5‐year PFS was 86.3% (95% confidence interval: 63.1–95.4), and 5‐year OS was 100% (Figure 1). The clinical course is summarized in Figure 2. Of the six symptomatic patients, four patients (two with abdominal pain and two with abdominal discomfort) were selected for the WW strategy, and two (one with abdominal pain and one with abdominal discomfort) were selected for rituximab therapy. Regarding treatment strategies, 23 (88%) patients were selected for the WW strategy, and three (12%) patients received the initial four cycles of rituximab therapy. Among the 23 patients selected for the WW strategy, 20 had SD, and three had PD (No. 8, No. 9, No. 21), including one with HT (No. 9). In the SD group, six had sCR, with median time from diagnosis to achieving sCR of 24.1 months (range: 11–95.7). Regarding the three patients with PD, two patients (No. 8, No. 9) were treated with chemotherapy and subsequently achieved complete response. In addition, one patient (No. 21) was continued with the WW strategy because of the low tumor burden despite disease progression.

Among the three patients treated with rituximab initially, two achieved complete responses, and one showed partial response. Among them, a relapsed patient (No. 4) with reappearance of duodenal lesion was selected for the WW strategy because of the low tumor burden and subsequently achieved sCR.

## 4 DISCUSSION

DFL is a rare and newly recognized disease [4]. There is no consensus about the treatment, and determining patients eligible for treatment is still debatable [9]. This study represents our single‐institution experience of DFL in which most of the patients were selected for the WW strategy with an annual follow‐up of EGD. The prognosis of patients with DFL is favorable, and some cases achieved sCR with the WW strategy. In addition, one patient achieved sCR with the WW strategy even after a relapse. The results were consistent with those of previous studies wherein various treatments were administered to patients. Therefore, the WW strategy under annual routine analysis may be an appropriate treatment option for DFL.

The prognosis of DFL is excellent. The largest retrospective study of DFL enrolled 125 patients with primary gastrointestinal FL, including 101 (89%) patients with duodenal involvement, in which more than half of the patients were treated with rituximab, cyclophosphamide, doxorubicin, vincristine, and prednisolone (R‐CHOP) or R‐CHOP‐like regimen or rituximab monotherapy and revealed that 5‐year PFS and 5‐year OS were 93% and 100%, respectively [6]. Another study showed that 3‐year PFS and 3‐year OS in 27 patients with DFL who received various treatments including the WW strategy and were followed up with EGD every 6 months were 70% and 100%, respectively [13]. Our study demonstrated that 5‐year PFS and OS were 86.3% and 100%, respectively, which is consistent with the results of previous reports [6, 13].

Since most studies on DFL are retrospective, no definite recommendations in terms of optimal treatment can be extrapolated [5, 13, 14, 15]. In general, four treatment strategies including the WW strategy, radiation therapy, immunotherapy, and immunochemotherapy are used for DFL, and the effectiveness of each strategy has been reported [5, 6, 13, 14]. The only prospective study on DFL including 29 patients with DFL demonstrated that there was no statistically significant difference in PFS or OS between the WW strategy and chemotherapy groups [15]. Considering that the OS rate was 100%, and the prognosis was excellent in the patients selected for the WW strategy, the authors concluded that the WW strategy was a desirable treatment strategy for DFL, although the number of patients was small [15]. Our study showed that the outcomes of DFL were favorable without initial treatment; therefore, the WW strategy was an appropriate treatment option.

From another perspective, some cases achieved sCR with the WW strategy [5, 13]. In the largest report on DFL management, including 63 patients, 24 patients underwent the WW strategy, and seven patients (29%) achieved sCR at a median follow‐up of 77 months [5]. In our study, among the 23 patients who were selected for the WW strategy, six patients (26%) achieved sCR at a median follow‐up of 65.5 months, and these results are comparable to those of the previous study [5]. Notably, the patient who was initially treated with rituximab relapsed and achieved sCR using the WW strategy. It was suggested that even at relapse, sCR could be achieved using the WW strategy in some patients. In other words, there is a group of patients for whom radiotherapy or chemotherapy could be harmful, and in such patients, the WW strategy might be beneficial.

In contrast, although relatively rare, patients with DFL have a risk of progression (<10%) and HT (3.8%) [4, 8]. Progression including HT could occur in DFL at any time, even after 5 years [8, 16]. Moreover, it is difficult to stratify prognosis using the international prognostic index or follicular lymphoma international prognostic index in DFL, and there are no methods to predict prognosis or progression in DFL [17]. Therefore, an appropriate long‐term follow‐up strategy is important in patients with DFL, especially those selected for the WW strategy, because it may help us to detect the progression and treat it appropriately. There is no evidence for the interval of follow‐up in patients with DFL [15], and there are various reports on follow‐up intervals ranging from 4 to 12 months [8, 13, 15, 16]. In our study, follow‐up included annual EGD, and we could detect progression (in four patients), and no deaths were attributed to DFL.

Our study has several limitations. First, there may be a bias of treatment selection and no definite treatment recommendations can be extrapolated because of the retrospective nature of this study. Second, the number of patients with DFL was small, and the treatment strategies were not compared. Third, we distinguished between FL and DFL by localization, not pathologically or genetically, which may be insufficient.

In conclusion, our study showed a very indolent clinical course and a good prognosis of DFL. The WW strategy for patients with DFL could be an appropriate and safe treatment option. However, in several cases, disease progression was documented, and regular follow‐up is important.

## CONFLICT OF INTEREST

The authors declare no conflict of interest.
